# Primary Pulmonary Alveolar Rhabdomyosarcoma in a Pediatric Patient: A Case Report With Literature Review

**DOI:** 10.7759/cureus.21270

**Published:** 2022-01-15

**Authors:** Bayan Hafiz, Hanaa Bamefleh

**Affiliations:** 1 Department of Anatomic Pathology, King Abdulaziz Medical City, Jeddah, SAU; 2 Department of Laboratory Medicine and Pathology, King Abdulaziz Medical City, Riyadh, SAU

**Keywords:** rhabdomyosarcoma, alveolar, lung, pleuropulmonary, pediatric, embryonal

## Abstract

Rhabdomyosarcoma (RMS) is a rare soft tissue tumor originating from skeletal muscle that is mostly reported in children. The most common sites of involvement are the head, neck, and extremities. The 2020 WHO classification divide RMS into four types: embryonal, alveolar, pleomorphic, and spindle cell/sclerosing. Reports of RMS with primary lung origin are rare. We present a case of RMS in a 16-month-old boy who presented with a lung mass and microscopic examination with fluorescence in situ hybridization confirmed the diagnosis of alveolar RMS. In conclusion, RMS should be considered in the differential diagnosis of any lung mass with small round blue cell morphology in the microscopic evaluation and should be distinguished from metastatic RMS of other sites, pleuropulmonary blastoma, lymphoma, neuroblastoma, primitive neuroectodermal tumor (PNET)/EWING, and malignant peripheral nerve sheet tumors (MPNST).

## Introduction

Rhabdomyosarcoma (RMS) is a malignant soft tissue neoplasm having skeletal muscle differentiation [1]. It is the most commonly occurring tumor in the pediatric age group and has a higher prevalence in males [1,2]. RMS is most frequently found in the head and neck area, followed by the genitourinary tract and extremities [3]. The World Health Organization (WHO) of soft tissue tumors has identified four subtypes: embryonal, alveolar, pleomorphic, and spindle cell/sclerosing [4]. The embryonal type is the most common type in children, with a favorable prognosis compared with other types [5]. Alveolar RMS has a high rate of metastasis and unfavorable prognosis; it is characterized by a chromosomal alteration - a fusion between the FKHR (also known as FOXO1) gene and either the PAX3 or PAX7 gene [6]. RMS rarely originates in the lung and only 32 such cases have been reported in the literature [7].

We report a case of a 16-month-old baby boy who presented clinically with shortness of breath and radiologically with a lung mass. The clinical presentation, radiological findings with pathology report, and fluorescence in situ hybridization (FISH) are compatible with primary alveolar RMS.

## Case presentation

A 16-month-old baby boy with known G6PD deficiency and club foot presented with progressive shortness of breath. His mother reported that the shortness of breath was associated with fever and decreased appetite. The baby had been delivered normally at full-term.

Physical examination revealed that the patient appeared ill and distressed. The vital signs were as follows: blood pressure, 100/70 mmHg; heart rate, 108 bpm; respiratory rate, 45 breaths/min; and O_2_ saturation, 89%. The chest examination revealed decreased breath sounds in the right chest. The remainder of the systemic review was unremarkable.

**Figure 1:**
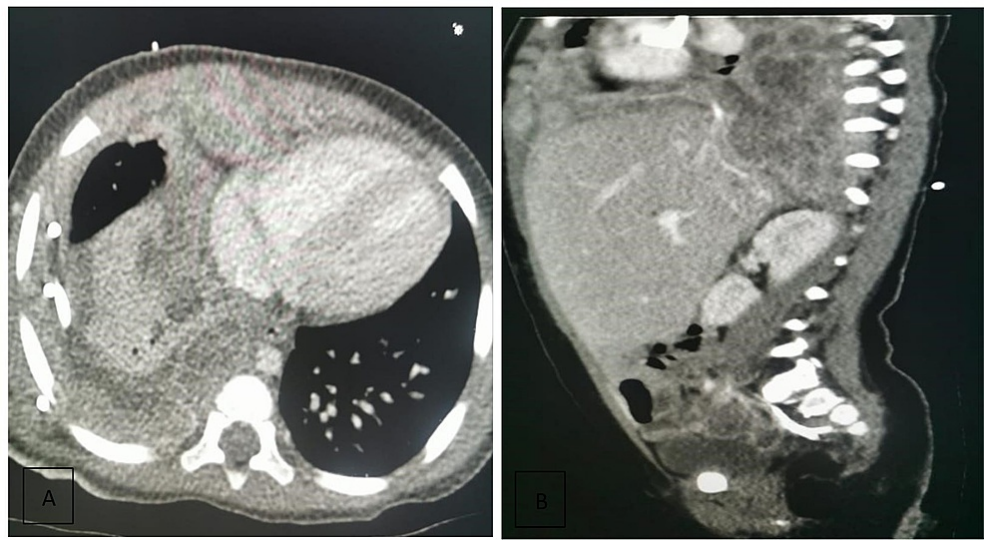
(A) Axial computerized tomography (CT) cut shows a lung base lobulated mass (7.3 x 6.4 x 4.4 cm). (B) A longitudinal CT cut shows the extension through the diaphragm.

Radiological studies, including computerized tomography (CT) and magnetic resonance imaging (MRI), were performed. The studies revealed a lobulated mass (7.3 x 6.4 x 4.4 cm) in the base of the right lung that involved the diaphragm, mediastinal pleura, and right pericardial space; the mass encased the esophagus and extended to the interlobular fissure. Three other pleural-based nodules were identified in the right upper lobe (Figures 1A, 1B). Based on the clinical and radiological findings, CT-guided core needle biopsies were obtained and sent for histopathology study.

**Figure 2:**
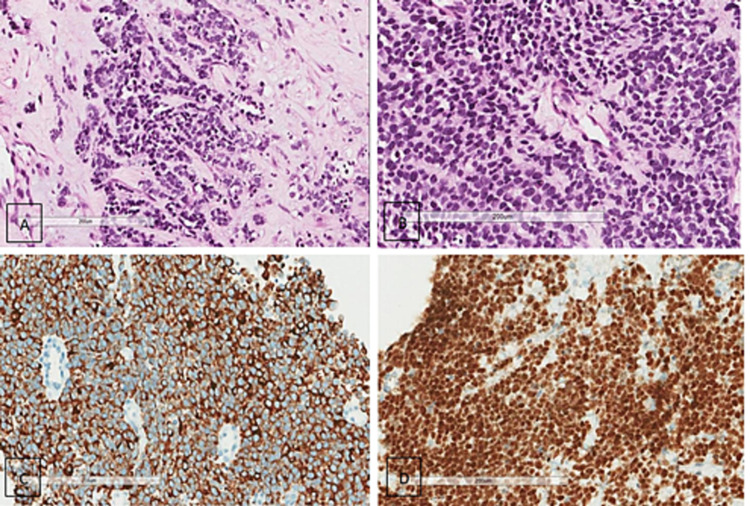
Histopathology examination with hematoxylin and eosin (H&E) stains and immunohistochemistry studies. Examination revealed neoplastic growth in the form of nests of small round blue cell tumors with some cells having a little amount of cytoplasm. (A) The nuclei were round with euchromatin. (B) Focal cytoplasmic striation was noted. Necrosis was rare (10x & 40x). (C) The desmin stain was diffuse positive with a membranous pattern. (D) MyoD1 showed nuclear positivity.

A pathological examination revealed six cores of lesioned tissue composed of nests of small round blue cell tumors, with some cells having little cytoplasm. The nuclei were round with euchromatin and focal cytoplasmic striation was noted. Necrosis was rare (Figures 2A, 2B). An immunohistochemistry panel was performed to label the following markers: desmin, pan-cytokeratin (CKpan), myogenin, synaptophysin, MyoD1, chromogranin, CD99, and CD45 S100. The tumor cells showed diffuse positivity for desmin, myogenin, MyoD1, and focal positivity for S100. The cells were negative for CD99, CKpan, CD45, chromogranin, and synaptophysin (Figures 2C, 2D). FISH revealed rearrangement of the FOXO1 gene at 13q14 (FOXO1 [13q14]), which is characteristic of alveolar RMS.

Based on the clinical history of no other primary in other sites of body and radiology in addition to microscopic features, immunohistochemistry, and the FISH study, the final diagnosis was primary pulmonary RMS, alveolar type. The patient received chemotherapy and radiotherapy for 10 months, demonstrating improvement at a follow-up imaging study. The patient showed complete remission at one-year post-treatment follow-up.

## Discussion

RMS is a primitive mesenchymal tumor with skeletal muscle differentiation. RMS is common in children and has a poor prognosis. Alveolar RMS has the worst prognosis due to its unique PAX3-FOXO1 fusion gene molecular phenotype [4].

RMS is associated with a congenital cystic adenomatoid malformation (CCAM) but may also occur in a healthy lung. The etiology of primary pulmonary RMS is still unknown, but there are two main hypotheses for its origin: first, the tumor may arise from heterotopic islets of striated muscle, which could explain the frequent association of RMS with pulmonary malformations such as cystic adenomatosis; and second, the tumor may arise from metaplastic changes in uncommitted mesenchymal cells in the absence of congenital abnormalities [3]. Like other lung neoplasms, RMS can present as a cough, respiratory distress, hemoptysis, chest pain, and/or recurrent pneumonitis [7]. Spontaneous pneumothorax has also been reported, especially in RMS cases that grow in the background of CCAM [8].

The main differential diagnosis is pleuropulmonary blastoma, lymphoma, neuroblastoma, primitive neuroectodermal tumor (PNET)/EWING, and malignant peripheral nerve sheet tumors (MPNST). Pleuropulmonary blastoma has blastema, anaplastic and epithelial components that are not present in RMS. The nuclear positivity for MyoD1 and myogenin is specific for RMS among other differential diagnoses.

An extensive search of English research literature (including PubMed, Google Scholar, and OVID) identified 32 cases reported as primary pulmonary RMS in the pediatric age group (Table 1) [8-32]. Fallon et al. diagnosed the first pediatric case of primary RMS in 1970 in a six-year-old girl [8]. Among the other cases, the ages of the patients ranged from five months to 16 years old. Nine of the cases developed in a background of CCAM, while the others-including our case-developed in a normal lung. Twenty-five of the cases were embryonal, two were pleomorphic, two were undifferentiated, and one had alveolar morphology. Our case represents the second reported case of alveolar RMS. Most patients received a chemotherapy regimen (vincristine, actinomycin, ifosfamide, and doxorubicin, in combination) according to the Intergroup Rhabdomyosarcoma Study (IRS) V protocol. Chemotherapy was combined with radiotherapy in several cases.

**Table 1:** Cases of primary pulmonary RMS CCAM: Congenital cystic adenomatoid malformation; RMS - Rhabdomyosarcoma

	Study	Age	Site	RMS Type	Treatment	Follow up
1	Fallan et al., 1970 [8]	6 years	Right bronchus	Embryonal	Chemotherapy and radiotherapy	Disease free to age 33
2	Udea et al., 1977 [9]	1-1/2 years	Left upper lobe, CCAM	Embryonal	Chemotherapy	Disease free to age 17
3	Krous and Sexauer, 1981 [10]	2-1/2 years	Left lower lobe	Embryonal	Chemotherapy and radiotherapy	Metastasis of brain and lymph node and death six months after diagnosis
4	Thomas et al., 1981 [11]	1 year and 9 months	Right-middle and lower lung	Embryonal	Chemotherapy	Disease free to age 5
5	Hartman and Shochat, 1983 [12]	11 years	Left main bronchus	Undifferentiated	Chemotherapy and radiotherapy	Free of disease 24 months after resection
6	Hartman and Shochat, 1983 [12]	13 years	Right side	Undifferentiated	Chemotherapy and radiotherapy	Disease free 5 years after the diagnosis and 1 year developed brain metastasis
7	Allan et al., 1986 [13]	2-1/2 years	Right lower lobe	Embryonal	Surgery and chemotherapy	Recurrent disease on the ipsilateral side 11 months after diagnosis
8	Allan et al., 1986 [13]	1 year and 9 months	Left lower lobe	Embryonal	Surgery and chemotherapy	Disease free after 4 years
9	Williams, 1986 [14]	1 year and 9 months	Right lower lobe, CCAM	Embryonal	Surgery and chemotherapy	Disease free to age 24
10	Shariff et al., 1988 [15]	1 year and 3 months	Left lower lobe, CCAM	Embryonal	Surgery only	Disease free to age 3
11	Hedlund et al., 1989 [16]	1 year and 10 months	Right side	Not recorded	chemotherapy	Disease free after 9 months.
12	Hedlund et al., 1989 [16]	1-1/2 years	Left upper lobe	Embryonal	chemotherapy	Disease free after 12 years
13	Murphy et al., 1992 [17]	3 years	Right middle lobe and right lower lobe, CCAM	Embryonal	Surgery and chemotherapy	Disease free to age 3
14	Murphy et al., 1992 [17]	3-1/2 years	Left lower lobe, CCAM	Embryonal	Surgery and chemotherapy	Disease free to age 6
15	Mcdermott et al., 1993 [18]	3 years	Right lower lung	Embryonal	Surgery and chemotherapy	Brain metastases and death
16	Mcdermott et al., 1993 [18]	2 years	Left side	Embryonal	Surgery and chemotherapy with radiotherapy	Died 5 months after intracerebral metastasis
17	Bogers et al., 1993 [19]	1-1/2 years	No information	No information	Chemotherapy	No information
18	Doval et al., 1994 [20]	10 years	Left main bronchus	Embryonal	Bronchoscopy with chemotherapy and radiotherapy	Disease Free
19	Noda et al., 1995 [21]	1 year and 10 months	Right upper lung	Alveolar	Surgery with chemotherapy and radiotherapy	Recurrence with brain metastasis after 6, 11, and 24 months and then complete remission till 5 years of age
20	d’Agostino et al., 1997 [22]	1 year and 10 months	Right lower lobe, CCAM	Embryonal	Surgery and chemotherapy	Disease Free to age 72
21	Ozcan et al., 2001 [23]	1 year	Left upper lobe, CCAM	Embryonal	Surgery and chemotherapy	Disease free to age 15
22	Iqbal et al., 2002 [24]	2 years and 8 months		Embryonal	Surgery and chemotherapy	Disease free 13 months after surgery
23	Doladzas et al., 2005 [25]	2 years	Left lower lobe, CCAM	Pleomorphic	No information	Disease free 10 years after diagnosis
24	Pia et al., 2005 [26]	2 years	Right lower lobe, CCAM	Embryonal	Chemotherapy pre- and post-surgery	Disease free to age 24
25	Chang et al., 2008 [27]	5 months	Right upper and middle lobes	Embryonal	Surgery and chemotherapy with proton beam radiation	Local recurrence after 24 weeks of treatment
26	Türkkan et al., 2010 [28]	12 years	Left lower zone	Embryonal	Chemotherapy followed by radiation	Died 9 months after the diagnosis
27	Lokesh et al., 2011 [29]	3 years	Right side	Embryonal	Chemotherapy	Disease free for 160 months
28	Lokesh et al., 2011 [29]	9 years	Right lower lobe	Embryonal	Chemotherapy	Disease free for 19 months
29	Lokesh et al., 2011 [29]	3 years	Right lower lobe	Embryonal	No chemotherapy or radiotherapy	Disease free for 7 months
30	Hassan et al., 2013 [30]	2 years	Left lower lobe	Embryonal	No information	No information
31	Balaji et al., 2016 [31]	9 years	Right lower lobe	Not determined	Chemotherapy	Disease free after 6 years
32	Mallapa et al., 2019 [32]	3 years	Left middle and lower zones	Embryonal	Chemotherapy	No information
33	Present case	1 year and 4 months	Right lower lung	Alveolar	Chemotherapy and radiotherapy	Disease free

## Conclusions

Primary pulmonary RMS is a rare disease that exhibits aggressive behavior. RMS should be included in the differential diagnosis of any lung mass with small round blue cell morphology. Clinical and radiological assessment is necessary to exclude metastatic RMS from other sites. In addition to RMS, other differential diagnoses that should be considered for a lung mass are pleuropulmonary blastoma, lymphoma, neuroblastoma, PNET/EWING, and MPNST.
